# Intraperitoneal extraosseous osteosarcoma: a case report and literatures review

**DOI:** 10.1186/s12891-020-03429-5

**Published:** 2020-07-11

**Authors:** Tiantian Wang, Shijie Liao, Xiaofei Ding, K. C. Anil, Qian Huang, Chengsen Lin, Jianming Mo, Haijun Tang, Yun Liu

**Affiliations:** 1 Departments of Orthopedics, The First Affiliated Hospital of Guangxi Medical University, Nanning, 530021 Guangxi China; 2 Research Centre for Regenerative Medicine, Guangxi Key Laboratory of Regenerative Medicine, Guangxi Medical University, 22 Shuangyong Road, Nanning, Guangxi China

**Keywords:** Osteosarcoma, Extraskeletal, Extraosseous, Intraperitoneal, Oft tissue osteosarcoma

## Abstract

**Background:**

To investigate the clinical imaging manifestations, diagnosis and treatment of intraperitoneal extraosseous osteosarcoma.

**Case presentation:**

A 52-year-old male patient with intraperitoneal extraosseous osteosarcoma was retrospectively analyzed. He suffered from left lower abdominal pain accompanied by mass for 6 months. On abdominal CT scan, multiple patchy and banded calcification were found. The largest is about six centimeters in diameter and underwent mass resection. Postoperative pathology revealed retroperitoneal osteosarcoma. The reported intraperitoneal extraosseous osteosarcoma was analyzed and the related literature was reviewed. Two years after operation, the patients had recurrence of the tumors and invaded sigmoid colon, peritoneum and bladder. Palliative operation was performed to remove the tumors in the bladder and transverse colostomy was performed. The patients were followed up by telephone and died 2 months after the second operation.

**Conclusions:**

Intraperitoneal extraosseous osteosarcoma has a low incidence and has no specific imaging features. Surgical resection is the main treatment and the prognosis is poor.

## Background

Extraosseous osteosarcoma, also known as soft tissue osteosarcoma, refers to osteosarcoma occurring in soft tissue rather than bone. Wilson [1] first described it in 1941.The incidence of extraosseous osteosarcoma is low, accounting for only 4% of osteosarcoma and 1% of soft tissue sarcoma [2]. At present, the treatment of soft tissue osteosarcoma mainly depends on surgical excision. The efficacy of adjuvant radiotherapy and chemotherapy is still controversial, and there is no consensus on the best combination of chemotherapy. Berner K [3] retrospectively analyzed the treatment of 37 patients with soft tissue osteosarcoma and found that there was no significant difference in 1-year survival rate between patients receiving targeted chemotherapy and palliative chemotherapy. After Torigoe [4] excised 20 patients with soft tissue osteosarcoma, a total of 15 patients received chemotherapy. Of these, 11 had no complete response, 5 had partial response, and 6 had no change or disease progression. The effective rate was 45%. They believe that treatments including systemic chemotherapy may improve the prognosis of patients with extraosseous osteosarcoma. At present, adjuvant chemotherapy after resection of soft tissue osteosarcoma is controversial, and the prognosis of soft tissue osteosarcoma is poor, metastasis and recurrence rate is higher [2, 3].

Therefore, it is necessary to summarize these cases. A case of peritoneal soft tissue osteosarcoma confirmed by pathology in our hospital was analyzed retrospectively and the related literature was reviewed. Objective: To investigate the clinical manifestation, diagnosis, treatment and prognosis of celiac osteosarcoma.

## Case presentation

**Fig. 1:**
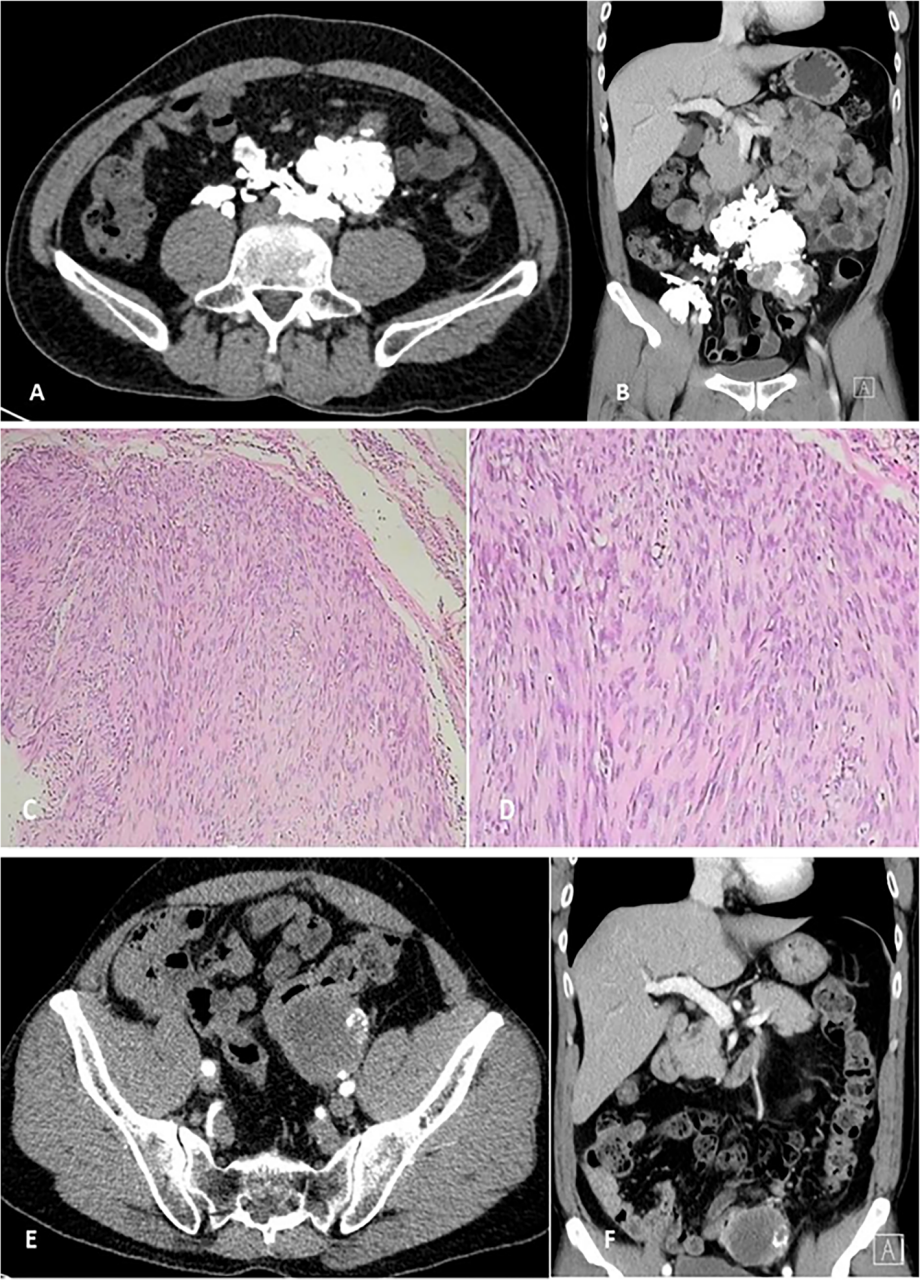
**a** and **b** preoperative CT: There were many irregular high and low mixed density shadows near the left lower abdomen and abdominal aorta, with clear demarcation and multiple patchy and banded calcification. The larger one was about 5.0 × 6.3. The enhanced lesions were unevenly enhanced. Pathology of (**c** and **d**) showed that spindle cell tumors were accompanied by a large number of hyaline degeneration and small focal ossification. **e**, **f** recurrence CT: There were a few masses, nodular high density lesions and soft tissue masses in the left lower abdomen. The largest size was about 4.8 *5.3 cm. On contrast-enhanced CT, uneven enhancement was seen, and nodular calcification was seen in them

A 52-year-old male was recurrent crampy left lower abdominal pain with mass. Symptoms had been present over 6 months. The mass of left lower abdomen was progressively enlarged. Physical examination: the left lower abdomen can touch the mass, about 5*6 cm in size, hard, no tenderness, clear boundary, slightly poor mobility, and no blood vessel pulsation. Abdominal CT: Multiple lesions and calcification in the lower abdomen. It is more likely to be considered as mesenteric cavernous hemangioma (Fig. 1a, b). Intraoperatively, the tumors originated from the posterior abdominal cavity and protruded to the left abdominal cavity. The tumors were hard as bones and irregular in shape. The 8 × 8 cm tumor was excised along the edge. Pathological findings: Spindle cell tumors with a large number of hyaline degeneration and small focal ossification. Immunohistochemistry: Vimentin (+), CD99 (+), CD117, CD34 and Dog-1 were all negative. They did not support gastrointestinal stromal tumors, Bcl-2 (−), SMA (−), NSE (−), S-100 (−), neurogenic tumors and solitary fibrous tumors, CK (−), epithelial tumors, and Ki-67 positive rate was about 15–20%. The diagnosis was osteosarcoma, chondroblast type (Fig. 1c, d). Patient were advised to receive chemotherapy after operation, but he refused. Two years after operation, abdominal CT showed a recurrence of tumors (Fig. 1e, f), which had invaded the colon and resulted in obstruction, infection and bladder invasion. Palliative surgery was performed to remove intravesical tumors and transverse colostomy. The patients were followed up by telephone and died 2 months after the second operation.

## Discussion and conclusions

### Clinical manifestation, diagnosis, treatment and prognosis of extraosseous osteosarcoma

Extraosseous osteosarcoma (EOS), also known as soft tissue osteosarcoma, is a rare malignant tumor that accounts for about 1% of all soft tissue sarcomas [2]. Preferred in the lower limbs, followed by the upper limbs, but also in the posterior peritoneum, iliac fossa, scalp, liver and other rare parts. EOS occurs in all age groups, but it is most common in middle-aged and elderly people around 50 years old. Males are slightly more than females, and the proportion is 1.9:1 [5]. Pathological subtypes of extraosseous osteosarcoma can be divided into osteoblasts, chondroblasts, fibroblasts, telangiectasis and small cell types [6].

The etiology of EOS remains unclear, but related risk factors include previous exposure to radiation, such as X-rays and radioactive thorium dioxide. Or at least 4 years after the development of the site that had previously received high-dose radiation therapy was induced to occur [6]. Some EOS reports occur after ossifying myositis [7]. In addition, 12 to 30% of patients have a history of trauma [8]. A case of extraosseous osteosarcoma occurring in the abdominal cavity has been reported [9]. A 61-year-old male patient had a history of abdominal pain for several years. Abdominal CT showed a mass about 10 cm in size. The tumors were found about 20 cm during the operation and were not resected. Pathological examination showed extraosseous osteosarcoma. Nine cycles of chemotherapy including doxorubicin, cisplatin and cyclophosphamide were given after operation. The tumors continued to progress and died 10 months later.

Typical clinical manifestation of Intraperitoneal extraosseous osteosarcoma is a progressively enlarged soft tissue mass. About one-third of patients suffer from occult onset and pain. Often, when patients go to treatment, the tumors grow very large [10]. The patient had a concealed onset, mainly with abdominal distension and abdominal mass. The abdominal mass was large when the patient came to the clinic.

The diagnosis of EOS must be combined with clinical manifestations, imaging and pathological examination. It should have the following points: (1) appearing in soft tissue and not adhering to bone or periosteum; (2) having a unified sarcoma pattern (excluding mixed malignant mesenchymal tumors); (3) producing bone-like and/or cartilage matrix [2].

It has been reported that extraosseous osteosarcoma is easily misdiagnosed as ossifying myositis, and later pathologically proved to be osteosarcoma [5]. The patient was initially diagnosed as mesenteric cavernous hemangioma, and pathologically diagnosed as solitary fibroma after ultrasound-guided puncture biopsy of lower abdominal mass. After the operation, the tumors were removed, frozen and immunohistochemically diagnosed as extraosseous osteosarcoma.

Radical or extensive resection, preoperative radiotherapy and adjuvant chemotherapy are the treatment options for extraosseous osteosarcoma. Expanding the scope of operation can only reduce the local recurrence rate, but can not prolong the survival time [5]. Active surgical treatment is useful for local recurrence control, but it does not reduce the incidence of death due to disease. Some scholars believe that traditional amputation of EOS can not improve the 5-year survival rate of patients, so limb salvage is recommended as far as possible [5]. At present, there are still disputes about the efficacy and regimen of EOS chemotherapy, and there are differences in the reported efficacy. Ahmad et al. [11] reported that 27 out of 60 EOS patients received doxorubicin-based chemotherapy with an effective rate of 19%. Doxorubicin is not effective in the treatment of EOS. However, the efficacy of preoperative and postoperative adjuvant chemotherapy for EOS is not clear, and there is no clear guidelines for adjuvant therapy. Radiotherapy is not a routine choice. Palliative treatment is given only when the patient is unable to undergo thorough surgical resection or tolerate high-dose chemotherapy or advanced disease. Van Rijswijk et al. [12] reported that radiotherapy can be used as palliative treatment to reduce the volume of tumors and improve the survival rate when the large soft tissue osteosarcoma of abdominal cavity can not be completely removed. Zhengfu Fan et al. [13] retrospectively analyzed 40 cases of localized high-grade extraosseous osteosarcoma. In multivariate analysis, radiotherapy improved the local recurrence-free survival rate, but did not improve the disease-specific survival rate. Adriamycin and isocyclophosphamide regimens were associated with local recurrence-free survival in stage III AJCC patients, but not with disease-specific survival. It can be seen that multimodal therapy, including adriamycin and isocyclophosphamide-based chemotherapy, radiotherapy and surgery, may be an effective treatment strategy for stage III diseases. Wakamatsu [14] demonstrated that adjuvant/neoadjuvant chemotherapy combined with extensive surgical resection could improve the prognosis of ESOS patients, and suggested that the chemotherapy regimen of adriamycin and isocyclophosphamide was superior to other ESOS regimens.

Regardless of its origin and location, the prognosis of EOS is poor, and there is no good treatment plan. Some scholars reported that tumor size less than 5 cm was an important factor affecting the prognosis of EOS. Bane reported that only 1 of 7 patients with tumors smaller than 5 cm died, but 14 of 16 patients with tumors larger than 5 cm died [15]. The tumor size of the patient in this case was about 8 cm, larger than 5 cm, and severe adhesion with surrounding tissues was observed during the operation, which was in the advanced stage of the disease. Two years after surgery, the prognosis is poor.

### The clinical manifestations, imaging features, recurrence rate and mortality of extraosseous osteosarcoma in abdominal cavity were reviewed

To review the relevant literature on intraperitoneal extraosseous osteosarcoma. The key words were “extraskeletal osteosarcoma”, “extraosseous osteosarcoma” and “soft tissue osteosarcoma”. “Intraperitoneal extraosseous osteosarcoma” The database of Pubmed. The literature is summarized in Table 1.

**Table 1:** Literature analysis of intraperitoneal extraosseous osteosarcoma

NO.	Author	Age	Sex	Tumor size (cm)	Histology type^a^	treatment	Local recurrence	Distant metastasis	Chemot-herapy	Radiation therapy	Status	Follow-up (m)
		(yrs)										
1	Heukamp [9]	61	M	20	1	Marginal	Yes	Yes	Yes	No	DOD	10
2	Lee [5]	37	F	10	2	lntral	Yes	No	No	No	DOD	64
3	Wakamatsu [14]	79	F	9	/	Marginal	/	/	No	No	DOD	2
4	WU [16]	39	F	6	2	Rad	/	Yes	Yes	No	DOD	8
5	Olgyai [17]	61	F	5	/	EL	Yes	Yes	Yes	No	DOD	10
6	Lee [18]	67	M	15	1	lntral	Yes	Yes	Yes	No	DOD	4

^a^1:osteoblastic; 2: chondroblastic; 3: fibroblastic; 4: telangiectatic; DOD: died of disease

Intral: intralesional; Margin: marginal excision; radical excision; EL:Exploratory laparotomy

Table 1 shows that the average age of onset of intraperitoneal extraosseous osteosarcoma is older. The clinical manifestations are generally not obvious. Surgical treatment is the main treatment, adjuvant chemotherapy and radiotherapy, and local embolization is also used. The average survival time after operation is short. Most of them died of advanced tumors such as lung metastasis associated with the disease. It can be seen that the survival time of 4 patients after chemotherapy is less than 1 year. Case 2 [5] survived for 64 months without chemotherapy and patient of our case survived for 2 years without chemotherapy. In these cases, chemotherapy does not seem to prolong the survival rate of patients with distant metastases who did not undergo radical resection. So further studies are needed to determine whether patients with distant metastases require routine chemotherapy.

In conclusion, extraosseous osteosarcoma has a high degree of malignancy and poor prognosis. Intra-abdominal extraosseous osteosarcoma is rare. Abdominal pain and abdominal mass are common clinical manifestations, but the early symptoms of the disease are often not obvious, and when found, the tumors are often very large. The imaging manifestation lacks specificity. It can be shown as one or more irregular high and low mixed density shadows with multiple patchy and banded calcification. The diagnosis of primary bone tumors in the skeletal system of the whole body should be excluded. The final diagnosis requires diagnostic laparotomy and pathological examination. Surgical excision is the most important treatment method, and chemotherapy and radiotherapy also have a certain effect. The size of tumors is one of the factors affecting prognosis.

## Data Availability

All the data needed to achieve the conclusion are presented in the paper.

## Abbreviations

EOS: Extraosseous osteosarcoma
CT: Computed Tomography
